# Evaluation of the effectiveness of a portable device for diagnosing cataract

**DOI:** 10.5935/0004-2749.2024-0249

**Published:** 2025-04-07

**Authors:** Camila Ribeiro Koch, Rafael Scherer, Liliane Souza Pereira, Tauanne Cândido, Milton Ruiz Alves, Newton Kara-Junior

**Affiliations:** 1 Department of Ophthalmology, Universidade de São Paulo, São Paulo, SP, Brazil; 2 Hospital Humberto Castro Lima, Salvador, BA, Brazil; 3 Vision Laser Palmas, Tocantins, TO, Brazil

**Keywords:** Cataract/diagnosis, Diagnostic techniques ophthalmological/instrumentation, Optical devices, Equipment and supplies, Eye-tracking technology

## Abstract

**Purpose:**

Access to cataract treatment and diagnostic tools continues to be hindered by financial and logistical barriers. Thus, photography-based cataract analysis via portable devices offers a promising solution for the detection of cataracts in remote regions. In this study, the accuracy of a portable device that is based on the Lens Opacities Classification III System for diagnosing cataracts was analyzed.

**Methods:**

Photographs of the anterior segment of the eye were taken in a low-light environment, and the pupillary region markings were automatically delineated using infrared photography. The captured images were automatically analyzed using a convolutional neural network. The study group included patients with cataracts, and the control group included patients without cataracts.

**Results:**

A total of 270 eyes were analyzed, which included 143 eyes with cataracts and 127 control eyes. A total of 599 photos were analyzed. The isolated nuclear cataract was the most frequently detected subtype (37.5%), followed by a nuclear cataract associated with a cortical cataract (30.3%). The device’s accuracy was 88.5% (Confidence intervals (CI), 83.19%-94.69%), specificity was 84.62% (CI 71.79%-97.30%), positive predictive value was 91.78% (CI 74.36%-97.30%), and negative predictive value was 82.50% (CI 74.36%-97.30%).

**Conclusion:**

The portable device is a simplified user-friendly cataract screening technique that can interpret results in remote regions. This innovation could mitigate the occurrence of cataract-induced blindness and prevent premature surgical interventions in early-stage cataracts.

## INTRODUCTION

Cataracts are a predominant cause of blindness globally. It is prevalent among individuals aged <50 years, affecting almost all those aged >80 years^(1)^. The most effective and widely used treatment for cataracts is phacoemulsification. However, the adoption of and accessibility to this procedure vary significantly across different regions, posing challenges in ensuring timely and affordable access to cataract surgeries^(2^-^4)^. Delays in the surgical treatment of cataracts reduce the patients’ quality of life and contribute to an increase in blindness worldwide.

Recent technological advancements, particularly in artificial intelligence (AI) and deep learning (DL) techniques, hold promise in enhancing the safety and efficiency of cataract screening. These innovations could drive down costs and streamline the diagnosis and treatment of cataracts, thereby improving accessibility to care. Although various automated platforms have been developed for this purpose, many fall short of the rigorous standards required for the accurate detection.^(5^-^8)^.

Seeking to improve the detection of cataracts by changing the crystalline lens color, a portable device was created that obtains photographs under controlled lighting conditions and focus^(9)^. Designed to detect cataracts using pupil images, this easy-to-use portable device could meet public health requirements, especially in resource-limited areas. Hence, in this prospective randomized study, we aimed to evaluate and validate the effectiveness of this device.

## METHODS

### Study population

In this prospective randomized study, we included adults with unilateral or bilateral cataracts who had undergone a clinical evaluation for cataracts at the *Hospital Humberto Castro Lima* in Salvador, Brazil, and at the Vision Laser Center for Visual Correction, Palmas, Tocantins, Brazil, between January 2023 and April 2023. The study was conducted in accordance with the guidelines of the Declaration of Helsinki, and it was approved by the Institutional Review Board of the Escola *Bahiana de Medicina e Saúde Pública* (No: 47826421.8.1001.5544; 05/15/2022). Informed consent was obtained from all the participants before enrollment. Study participants were selected via a draw using a table of random numbers that were based on sequential numbering. The inclusion criteria for the cataract group were individuals aged >18 years with cataracts. The control group comprised individuals aged >18 years old without cataracts. The exclusion criteria for both groups were participants with pseudophakic eyes, ocular trauma, uveitis, or age <18 years. The control group consisted of companions of the patients being treated during the study period. The cataract subtypes evaluated in this study were isolated nuclear cataracts, cataracts associated with cortical or posterior subcapsular cataracts, and isolated cortical cataracts. Isolated posterior subcapsular cataracts were not evaluated because the evaluation equipment used lacked a retro illumination mode that is required to diagnose this cataract subtype.

### Patient data

The study participants underwent a complete eye examination on time. Two ophthalmologists were responsible for the ophthalmological evaluation, which included visual acuity (VA) with the Snellen chart, cycloplegic refraction, slit-lamp biomicroscopy, appla-nation tonometry (Goldmann), and dilated fundus biomicroscopy. Under slit-lamp examination, the lens opacity in the dilated eye was graded according to the Lens Opacities Classification System III (LOCS III)^(10^,^11)^. The LOCS III is the gold standard for classifying cataracts and consists of using six slit-lamp images for grading the nuclear color (NC), nuclear opacity (NO), as well non-isolate nuclear cataracts or isolate cortical cataracts. NC and NO grades range from 1 to 6, and they are directly related to the cataract intensity^(11)^.

The control group only included patients with LOCS III NO 0, NC 0, C 0, and P 0. In case of disagreements between the ophthalmologists, a third ophthalmologist acted as a reviewer. The following patient data were also collected: age, sex, and corrected distance VA (CDVA).

### Image captured

**Figure 1:**
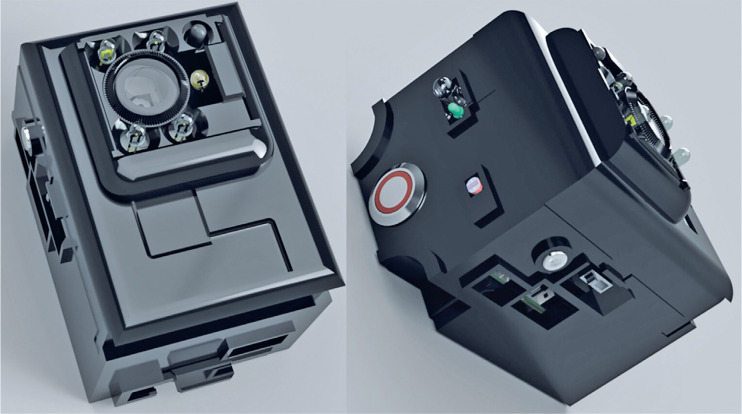
Portable device used to capture images of the lens.

Images of the anterior segment of the patients and controls were obtained with the portable device in study. At least two subsequent eye images (and measurement) were taken while repositioning the participant between each shot. This was done to obtain images in different angles. The images (and measurements) were obtained in a room without sunlight or artificial lighting. All images were obtained using the same device. The portable equipment (Figure 1) used to capture the images is a patented device registered with the National Institute of Industrial Property (Reg No: BR 10 2017 023842 3). It allows photography under controlled lighting conditions and lens focus, provides markings of the pupillary region, measures parameters related to the color of the region, and automatically analyzes these parameters. The markings on the pupil are identified using infrared photography and transposed to sequential photographs with white light. The device utilizes an 8-inch megapixel camera to capture lens reflections and employs a microprocessor to differentiate between the lens reflection, corneal reflection, and pupillary region. In 2022, Scherer et al. published an article on the patented equipment and provided comprehensive insights into its specifications^(9)^.

### Data analysis

**Figure 2:**
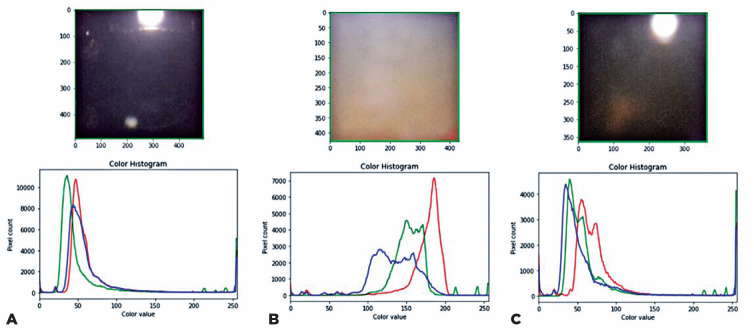
Regions of interest in three patients. The pupillary regions of A) a patient without a cataract (on the left), B) a patient with an LOCS III NO6 NC6 cataract (in the center), and C) a patient with an LOCS III NO3 NC3 cataract (on the right) and their respective color histograms are depicted.

To analyze the captured images, a DL algorithm was developed using Python (Python Software Foundation, USA) and TensorFlow (Google Inc., USA). Figure 2 illustrates how the device analyzes the different opacity densities in the lens nucleus.

A combination of machine learning (ML) and transfer learning was used with DenseNet-121, which had been pretrained with the ImageNet database. The model was trained using 25 epochs, with a final training loss of 0.48, test loss of 0.43, and batch size of 32. The training set of 486 images and test set of 113 images were divided randomly at the patient level (80%/20%). Training was performed with 25 epochs to avoid overfitting because of the lack of a validation set due to the restricted sample size. The adaptive moment estimation (ADAM) was used as the optimizer. The images that had been already resized to 256 x 256 pixels were randomly flipped horizontally to augment the training dataset; these were the only preprocessing methods applied.

### Statistical analysis

To analyze the level of statistical significance, the bootstrap method was used with 10,000 interactions. Clinical significance was set at p<0.05. To analyze the agreement of measurements in the subsequent images of the same eye in the same patient, Cohen’s kappa coefficient was used. The sensitivity, specificity, accuracy, positive predictive value, and negative predictive value of the classification determined by the device were compared to those of the LOCS III classification determined by ophthalmologists. Confidence intervals (CI) were calculated using the Clopper-Pearson method for sensitivity, specificity, and accuracy, and the Mercaldo et al.^(12)^ method for positive and negative predictive values. The effectiveness will be evaluated on the basis of classification accuracy and supported by the sensitivity, specificity, positive predictive value, and negative predictive value.

## RESULTS

A total of 270 eyes (153 patients) were included in the study. Of these, 143 eyes were included in the cataract group and 127 eyes were included in the control group. The mean age was 68.1 years in the cataracts group and 44.9 years in the control group (p<0.0001). The proportion of females were higher than those of men in both groups (p=0.0176). The isolated nuclear cataract (37.5%) was the most frequently detected cataract subtype, followed by nuclear cataract associated with cortical cataract (30.3%) and nuclear cataract associated with posterior subcapsular cataract (10.5%). Of the patients with nuclear cataract, 37.5% were classified as LOCS III N0/N1, 32.3% as LOCS III N2, and 30.3% as LOCS III N3. Table 1 includes the demographic data and visual acuity of the study participants.

**Table 1:** Demographic data and visual acuity of the cataract and control groups

	Mean age (years), n (max, min)	Sex n (%)	Visual acuity as per Snellen chart, n (%)		
			£ 20/40	>20/40 and £ 20/200	>20/200
Cataract	68.1 (49, 88)	Female: 60 (57.4) Male: 45 (42.6)	50 (34.9)	72 (50)	22 (15.1)
Control	44.9 (18, 82)	Female: 37 (75.4) Male: 12 (24.6)	123 (97)	1 (0.7)	3 (2.2)

### Image analysis

**Figure 3:**
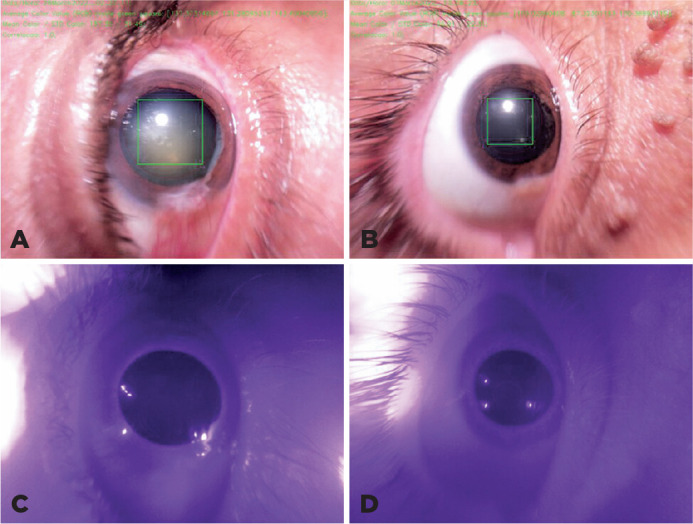
Images obtained using the patented equipment that depict the marking of the region of interest in A) a patient with an LOCS III NO3 NC3 cataract (on the right) and B) a control without a cataract (on the left). The corresponding infrared images for pupil marking in C) the patient with cataract and D) are also depicted.

The cataract was analyzed on the basis of the density of the lens nucleus opacification. Figure 3 depicts an example of how the software differentiates between a patient with a cataract and a control who does not have a cataract.

**The sum of patients in both groups is greater than the total by 1 unit as one patient had a cataract in one eye and no cataract in the other, resulting in an overlap between the groups.

**Figure 4:**
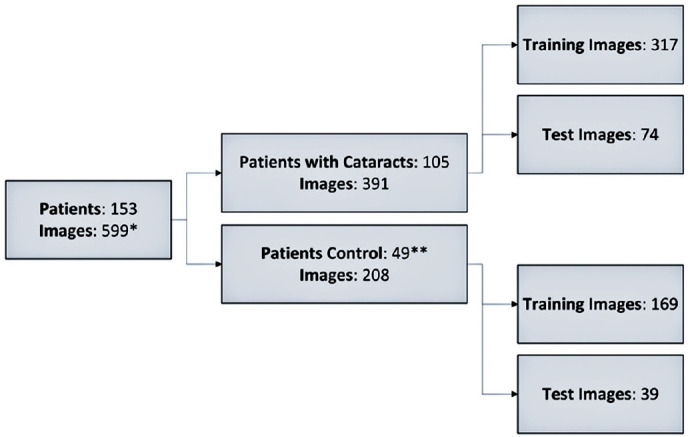
Classification of the patient sample in the study.

A total of 949 images were captured in the study, and in 599 (63.1%) of them, the equipment was able to accurately identify the region of interest (pupillary area). Of the 559 images, 391 were taken from 105 patients with cataracts, and 208 were from 49 control patients. The average number of captures per patient eye was 2.71 images (SD, ± 1.06). Figure 4 depicts the classification of the patient sample in the study.

### Artificial intelligence

Considering the best cutoff point calculated using the Youden index with the training set (52.91%), which are images assigned a value equal to or greater than the cutoff (Graphic 1), the AI-derived metrics for cataract diagnosis in the test set were calculated (20% of the research sample). The accuracy of the device was 88.5% (CI, 81.3%-93.7%), specificity was 84.62% (CI, 69.5%-94.1%), sensibility was 90.5% (CI, 81.5-96.1), positive predictive value was 91.78% (CI, 84.2%-95.9%), and negative predictive value was 82.50% (CI, 69.7-90.6).

**Graphic 1:**
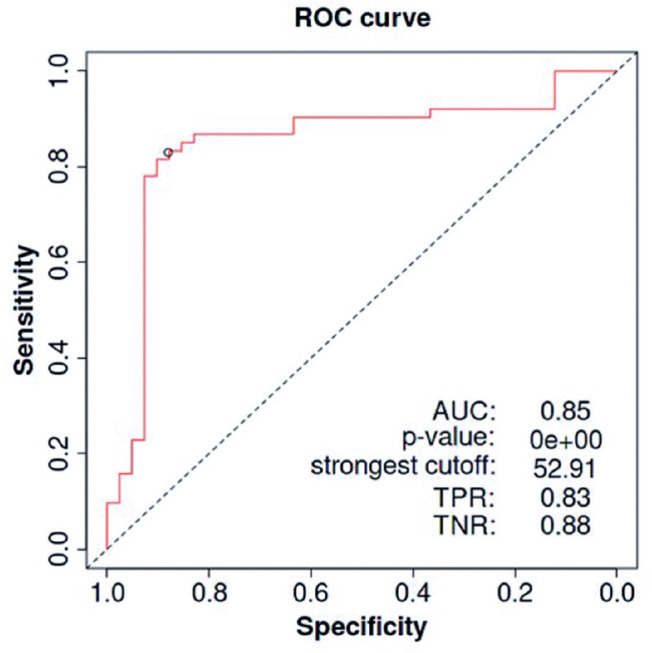
ROC curve of the artificial intelligence analysis method applied to the Regions of Interest extracted from the equipment’s captures to classify cataracts or the absence of cataracts.

The Cohen’s kappa coefficient for two consecutive measurements of the same eye was 0.83, which indicates an almost perfect agreement. Furthermore, the average difference between the measurements was only 12.85% (SD, ± 15.12%).

## DISCUSSION

The pursuit of enhancing a patient’s quality of life is continuous, particularly as life expectancy continues to rise. Recognizing the direct impact of cataracts on the quality of life and their prevalence highlights the need for easily accessible, accurate, and cost-effective methods of cataract detection. Moreover, it is essential to utilize a robust and enduring platform that remains impervious to the continuous flux in the components and equipment specifications^(13)^. This approach safeguards against any degradation in the accuracy of technologies that is reliant on computers. This study highlights the outcomes of a device that aligns with these specifications.

The device in this study is unique because it uses a camera without an infrared filter for measurements. The device was used to measure several parameters in addition to the amount of reflected light. The device optimizes its portability as well as the simplicity with which it displays results. Therefore, the proposed device can be used even by those with a minimum level of education in remote regions to screen patients before they are evaluated by a specialized ophthalmological.

AI first emerged in 1956, followed by ML, highlighting the ability of computers to learn without explicit programing. ML allows algorithms to make predictions based on training data, while DL enables models to automatically extract complex features via deep neural networks. Thus, DL offers higher accuracy and efficiency in tasks such as image recognition and classification. Especially in ophthalmology, DL has demonstrated promise in detecting diseases from medical imaging studies such as fundus photography and optical coherence tomography^(14)^. To improve the efficacy of the device and ensure continued improvement, we integrated AI and ML. This demonstrates that the device discussed in the study is compatible to those that will be used in ophthalmology in the future.

A technology using AP from a national cataract screening program in China, ResNet, was trained with 37,.638 images. In the study, the area under the ROC curve was >91% for the diagnosis of referable cataract^(15)^. Zhang et al.^(16)^ also used ResNet and fundus imaging to classify cataracts into six distinct groups, and they achieved an average accuracy of 92.66%. However, the accuracy of measurements in poor-quality images was limited. In another study, the Retinal Artificial Intelligence Diagnosis System achieved a sensitivity of 89.8% and accuracy of 95.3% to 99.9% in 208,758 images. In our study, the portable device exhibited an accuracy of 88.5% (81.3-93.7) and sensitivity of 90.5% in 949 captured images, demonstrating significant and promising results. The classification determined using the device photographs were compared to the LOCS III classification determined via slit-lamp examination, which further provides credibility to our results. One limitation of this device was its ability to analyze only nuclear cataracts.

In conclusion, the automated, portable, easy-to-use device that was evaluated in our study can be used for identifying cataracts that require surgery. Future studies should be aimed at enhancing the outcomes across all cataract classifications and age groups and refining the product design. This is plausible because the performance can be constantly improved with ML. New devices that utilize AI present challenges for validation, clinical implementation, and future recommendations. This device presents a promising solution to the myriad challenges currently faced by ophthalmologists and healthcare professionals worldwide.
